# A Nonlinear Adaptive Beamforming Algorithm Based on Least Squares Support Vector Regression

**DOI:** 10.3390/s120912424

**Published:** 2012-09-12

**Authors:** Lutao Wang, Gang Jin, Zhengzhou Li, Hongbin Xu

**Affiliations:** 1 School of Automation Engineering, University of Electronic Science and Technology of China, Chengdu 611731, China; E-Mail: hbxu@uestc.edu.cn; 2 China Aerodynamics Research & Development Center, Mianyang 621000, China; E-Mail: gjin@ioe.ac.cn; 3 School of Communication, Chongqing University, Chongqing 400044, China; E-Mail: lizhengzhou@cqu.edu.cn

**Keywords:** adaptive beamforming, least-squares support vector regression (LS-SVR), sparsification, kernel function

## Abstract

To overcome the performance degradation in the presence of steering vector mismatches, strict restrictions on the number of available snapshots, and numerous interferences, a novel beamforming approach based on nonlinear least-square support vector regression machine (LS-SVR) is derived in this paper. In this approach, the conventional linearly constrained minimum variance cost function used by minimum variance distortionless response (MVDR) beamformer is replaced by a squared-loss function to increase robustness in complex scenarios and provide additional control over the sidelobe level. Gaussian kernels are also used to obtain better generalization capacity. This novel approach has two highlights, one is a recursive regression procedure to estimate the weight vectors on real-time, the other is a sparse model with novelty criterion to reduce the final size of the beamformer. The analysis and simulation tests show that the proposed approach offers better noise suppression capability and achieve near optimal signal-to-interference-and-noise ratio (SINR) with a low computational burden, as compared to other recently proposed robust beamforming techniques.

## 1. Introduction

As one important branch of modern array signal processing, the beamforming technique has been widely studied and applied in the radar, wireless communication, sonar, medical imaging, as well as astronomy domains. The standard beamforming approach, such as the minimum variance distortionless response (MVDR) beamformer [1], was usually established based on an ideal antenna array with exactly known array manifold. Thus, it is very sensitive to practical circumstances, and its performance would be seriously degraded by diverse factors, such as the steering vector mismatch, array calibration errors and snapshot number restrictions.

During the last decades, in order to resist the model mismatches and possible environment changes, the robust beamforming approach have been largely studied [2–5]. Among others, by introducing a penalty term into the objective function, the diagonal loading (DL) algorithm could effectively reduce the eigenvalue spread of the noise and prevent the distortion of beampattern [6]. Nevertheless, how to get the optimal loading factor for DL is still a serious issue when the desired steering vector and/or the available snapshot numbers are uncertain [7]. A robust adaptive beamforming, based on the worst-case performance optimization, would delimit the uncertainty set of steering vectors by upper bounding the norm of the steering vector mismatch [8]. However, neither the mismatch vector nor its upper bound is known in practice. To overcome this model defect in standard DL algorithm, an adaptive beamforming method was developed, which estimates iteratively the difference between the actual and presumed steering vectors in order to maximize the output signal-to-noise plus interference ratio (SINR) [9–11]. But this adaptive beamforming algorithm is not sufficiently reliable in the case when the snapshots are small.

In order to reject jamming signals, poor array calibration, signal wave-front distortions, the minimum-variance-distortionless-response (MVDR) beamforming is modified by the means of incorporating multiple linear constrains [12–14]. Whereas, the augmentation of constrains would reduce the array freedom degrees in the linear beamforming framework. Nonlinear beamforming approaches provide a novel idea to address this issue for they can adapt better to the statistical properties of the given data than linear ones [15]. Neural network has been applied to beamforming among other nonlinear array processing tasks. But this approach suffers from serious drawbacks such as over-fitting or local minima, which leads to suboptimal solutions [16].

Support Vector Machines (SVM), introduced by Vapnik [17], is an important new methodology for pattern classification and nonlinear function approximation. This method addresses the beamforming problem by means of incorporating additional inequality constrains to penalize sidelobe levels and allowing a certain error in the desired signal direction [18]. Thus the MVDR beamforming method is reformulated and the cost function turns out to be equivalent to SVM for regression. However, the time consumed to train SVM beamformer scales super linearly to the number of observations, and it leads to an insurmountable computational burden in online operation modes [19]. The least-squares support vector machine (LS-SVM) inherits the SVM's generalization capacity. By solving linear equations instead of a quadratic programming (QP) problem in the standard SVM, the training procedure and the computational complexity of the standard SVM would be effectively simplified [20]. The main drawback of LS-SVM is that it works in batch mode. Thus, it is difficult to be used in large-scale applications. Recent researches about LS-SVM continuously focus on the improvement of the training algorithms, model selection and sparseness [21,22].

This paper presents a new LS-SVR-based approach to address the robust beamforming issue. This approach alleviates the array output SINR degradation in the presence of steering vector mismatches, strict restrictions on the number of available snapshots, and numerous interferences by replacing the conventional linearly constrained minimum variance cost function with a squared-loss function, and achieves better generalization capacity by applying Gaussian kernels to the array observations. We also present a fast recursive procedure to estimate the weight vectors on real-time, and a novelty criterion to perform model reduction. The paper is organized as follows. The signal model, also the minimum mean square error (MMSE) and the MVDR-beamformer solutions are presented in Section 2. The basic principle of LS-SVR-based beamforming method is introduced in Section 3. In Section 4, a recursive procedure to calculate the regression parameters is provided. And a sparse mode is presented in Section 5. The simulation tests under different mismatch scenarios are illustrated in Section 6. A summary conclusion is given at the last of this paper.

## 2. Sensor Signal Model

Consider a linear array of M sensors receives signals from D narrowband source. The vector of array observations x(*t*) ∈ C*^M^*^×1^ at time *t* could be modeled as:

(1) x(t)=As(t)+n(t)

where, **θ** = [*θ*_1_, *θ*_2_,…, *θ_D_*]^T^ ∈ R*^D^*^×1^ is the vector with the directions of arrival (DOA) and (·)*^T^* stands for transpose, **A** = [**a**(*θ*_1_), **a**(*θ*_2_)…**a**(*θ_D_*)] ∈ **C***^M^*^×D^ is the matrix containing the array steering vectors **a** (*θ_i_*) = [1,*e*^−^*^j^*^2^*^π^*^sin(^*^θi^*^)^*^d^*^/^*^λ^*…*e*^−^*^j^*^2^*^π^*^sin(^*^θi^*^)^*^d^*^/^*^λ^*]*^T^*. The uncorrelated sources are represented by the vector *s*(*t*) = [*s*_1_(*k*), *s*_2_(*k*))…*s_D_*(*k*)]*^T^* ∈ **C***^M^*^×1^. The vector **n**(*t*) ∈ **C***^M^*^×1^ is the sensor noise, and it is assumed as complex Gaussian with zero-mean:

(2) $$A=\left[a\left(\theta_{1}\right),a\left(\theta_{2}\right)\cdots a\left(\theta_{D}\right)\right]$$

The output of the beamformer is defined as:

(3) $$y(t)={\text{w}}^{H}\text{x}(t)$$

where, **w** = [*w*_1_, …, *w_M_*]^T^ ∈ C*^M^*^×1^ is the complex vector of beamformer weights, (·)^T^ denotes transpose, and (·)^H^ denotes Hermitian transpose.

If certain observations are known during the procedure of training parameters, then, according to the MMSE criterion, the complex vector of beamformer weights **w** can be described as:

(4) $$\text{w}={\text{R}}^{-1}\text{p}$$

where, **R** is *M* × *M* covariance matrix, and **p** is the cross-correlation between the desired output and the received signal.

The classical MVDR beamformer minimizes the array output energy, and the weights subject to a constraint of unity array response on the desired array steering vectors, that is:

(5) $$\begin{array}{ccc} \underset{w}{\text{min}\;}w^{\text{H}}Rw & \text{s}.\text{t}. & w^{\text{H}}a(\theta_{1})=1 \end{array}$$

The constraint **w**^H^**a**(*θ*_1_) = 1 prevents the gain at the look direction from being reduced, and the solution of Equation (5) can be easily estimated by means of using Largrange multiplier method:

(6) $$w=\frac{R^{-1}a(\theta_{1})}{a{\left(\theta_{1}\right)}^{\text{H}}R^{-1}a(\theta_{1})}$$

In practice, it is not feasible to calculate the exact covariance matrix **R** and it would be estimated by the sample covariance matrix $\hat{R}=\frac{1}{K}\overset{K}{\underset{k=1}{\text{∑}}}x\left(k\right)x^{H}\left(k\right)$ where *K* is the number of observed snapshots.

The performance of MVDR beamformer in Equation (5) is sensitive to mismatch between the presumed and actual steering vectors due to the uncertainty of the desired signal DOA, strict restrictions on the number of available snapshots, and numerous interferences.

## 3. LS-SVR-Based Beamforming Method

### 3.1. Nonlinear SVM-Based Beamforming

Consider a set of snapshots **x***_i_*, *i* = 1, *N* at time *t* from an array and the corresponding set of desired symbols **y***_i_*, *i* = 1, *N*, are available for training purpose. The basic idea of nonlinear beamforming is to transform the data set **x***_i_*, *i* = 1, *N* into a higher (possibly infinite) dimension feature space H by a nonlinear transformation *φ*(·). Thus, the beamformer's output can be formulated as a linear regression in H. It could be expressed as:

(7) $$y_{i}=w^{\text{H}}\phi (x_{i})+e_{i}$$

where, **w** ∈ H is the linear parameter set and *e_i_* is the output error.

The parameter set **w** can be estimated by minimizing a certain cost function on output error *e_i_*. For SVM regression, the parameter set **w** and the *ε*–intensive loss function could be estimated by the minimum risk criterion, *i.e.*,

(8) $$\text{min}J(w,ɛ)=\frac{1}{2}{\left\|w\right\|}^{2}+C\sum_{i=1}^{N}L_{ɛ}(\xi_{i},\zeta_{i})$$

subject to *ξ_n_*, *ξ_n_* ≥ 0. Where, *C* ≥ 0 is the tradeoff term between the minimization of the weight norm and the output error. The *ε*–intensive loss function is given by:

(9) $$L(e)=\{\begin{array}{ll} 0, & \left|e\right|<ɛ\\ \left|e\right|-ɛ, & \left|e\right|\geq ɛ \end{array}$$

where *ε* is a positive parameter which is used as an error threshold.

The weight vector **w** is regularized by solving Equation (8), Thus, the generalization capacity of the beamformer will be remarkably improved.

### 3.2. Nonlinear LS-SVR Beamforming

Instead of the inequality constrains in standard SVM algorithm, the equality ones are taken in LS-SVR, and the linear equation of the *ε*–intensive loss function is replaced by a quadratic equation. Therefore, The LS-SVR beamformer can be described as the following quadratic optimization problem [20]:

(10) $$\begin{array}{c} \text{min}\;J({\bar{w}}_{t},{\bar{e}}_{t})=\frac{1}{2}{\left\|{\bar{w}}_{t}\right\|}^{2}+C\sum_{i=1}^{N}e_{i,t}^{2}\\ \;\text{s}.\text{t}.\;e_{i,t}={\bar{y}}_{i,t}-{\bar{w}}_{t}^{\text{T}}\phi ({\bar{x}}_{i,t})-b_{t},i=1,2,\cdots ,N \end{array}$$

where, $e_{t}^{i}$ is the error at time *t*. The sum of squared errors in Equation (10) represents the *ε*–intensive loss function under the linear constraint. This treatment would greatly reduce the computation complexity since only the linear equation, instead of the QP problem in SVM, is solved.

The array observations of the beamformer are complex, whereas the variables in the objective function of SVM are real. So, it is necessary to rewrite the complex variables as real variables. For this reason, the array observations **x***_i_*, the beamformer outputs *y_i_* and the weight vectors **w***_t_* are rewritten as:

(11) $$\begin{array}{cc} {\bar{x}}_{i,j} & =\{\begin{array}{c} {\begin{array}{ll} {}[\text{Re}(x_{i,t}^{\text{T}}) & \text{Im}(x_{i,t}^{\text{T}})] \end{array}}^{\text{T}}\begin{array}{cc} \in R^{2M}, & i=1,\cdots ,N \end{array}\\ {\begin{array}{ll} {}[\text{Im}(x_{i-t,t}^{\text{T}}) & -\text{Re}(x_{i-t,t}^{\text{T}})] \end{array}}^{\text{T}}\begin{array}{cc} \in R^{2M}, & i=t+1,\cdots ,2N \end{array} \end{array}\\ {\bar{w}}_{t} & ={\left[\begin{array}{cc} \text{Re}(w_{t}^{\text{T}}) & \text{Im}(w_{t}^{\text{T}}) \end{array}\right]}^{\text{T}}\in R^{2M}\\ {\bar{y}}_{i,j} & =\{\begin{array}{ll} \text{Re}(y_{i,t}), & i=1,\cdots ,N\\ \text{Im}(y_{i-t,t}), & i=t+1,\cdots ,2N \end{array} \end{array}$$

The result of the quadratic optimization problem of Equation (10) is the saddle point of the following Lagrange function:

(12) $$L({\bar{x}}_{i,t},b_{t},{\bar{e}}_{t},\alpha_{t})=J({\bar{w}}_{t},e_{t})-\sum_{i=1}^{2\text{N}}\alpha_{i,t}\{{\bar{w}}_{t}^{\text{T}}\phi ({\bar{x}}_{i,t})+b_{t}+e_{i,t}-{\bar{y}}_{i,t}\}$$

where, **α***_t_* = (*α*_1_, *α*_2_, … *α*_2_*_N_*)^T^, *α*_i_ > 0 is Lagrange multipliers, defined as regression parameters in this paper.

According to the Karush-Kuhn-Tucker (KKT) conditions, differentiating the above function with respect to the Lagrange multipliers **α***_t_* and **x̄***_i,t_*, *b_t_*, *e_i,t_* yields:

(13) $$\left\{ \begin{array}{l} \frac{\partial L}{\partial {\bar{w}}_{t}}=0\Rightarrow {\bar{w}}_{t}=\sum_{i=1}^{2N}\alpha_{i}\phi ({\bar{x}}_{i,t})\\ \frac{\partial L}{\partial b_{t}}=0\Rightarrow \sum_{i=1}^{2N}\alpha_{i}=0\\ \frac{\partial L}{\partial {\bar{e}}_{i,t}}=0\Rightarrow \alpha_{i}=Ce_{i,t}\\ \frac{\partial L}{\partial \alpha_{i,t}}=0\Rightarrow {\bar{w}}_{t}^{\text{T}}\phi ({\bar{x}}_{i,t})+b_{t}+e_{i,t}-y_{i,t} \end{array}\right.$$

The system obtained from the KKT conditions is linear. Its result is obtained by solving the linear system which is expressed as following matrix:

(14) $$\left[\begin{array}{cc} 0 & {\bar{e}}^{\text{T}}\\ \bar{e} & Q_{t}+C^{-1}I \end{array}\right]\;\left[\begin{array}{c} b_{t}\\ \alpha_{t} \end{array}\right]=\left[\begin{array}{c} 0\\ {\bar{y}}_{t} \end{array}\right]$$

where, **ȳ***_t_* = (*ȳ*_1_, *ȳ*_2_ … *ȳ*_2_*_N_*)^T^, **ē** = (1,1,…,1;^T^, **Q***_i_* is Gramm matrix and the element of **Q***_i_* is **Q***_i,j_* =< *φ*(**x̄***_i,j_*), *φ*(**x̄***_i,j_*) >= *k_t_*(**x̄***_i_*, **x̄***_j_*), *i*,*j* = 1,2,…2*N*, *k_t_*(**x̄***_i_*, **x̄***_j_*) denotes kernel function responsible for the nonlinear mapping *φ*(&moddot;), which greatly simplify the inner product calculation in the feature space. Thus, linear methods can be applied on the transformed data, and it is not necessary to perform computations in the high-dimensional feature space. As the most widely used kernel function in many practical applications, Gaussian kernel is taken here:

(15) $$k({\bar{x}}_{i},{\bar{x}}_{j})=\exp\left(-\frac{{\left\|{\bar{x}}_{i}-{\bar{x}}_{j}\right\|}^{2}}{2\sigma^{2}}\right)$$

where σ > 0 is the kernel radius.

The outputs of the nonlinear LS-SVR beamformer are:

(16) $${\bar{y}}_{t+1}=\sum_{i=1}^{2N}\alpha_{i}k({\bar{x}}_{t+1},{\bar{x}}_{i,t})+b_{t}$$

## 4. Recursive Algorithms

From Equation (16), it could be known that once the regression parameters **α***_t_* and *b_t_* are computed, the beamformer outputs can be obtained. Denoting **U***_t_* = **H***_t_*^−1^ = (**Q***_t_* + *C*^−1^**I**)^−1^, the result of LS-SVR (Equation (14)) can be represented as:

(17) $$\left[\begin{array}{cc} 0 & {\bar{e}}^{\text{T}}\\ \bar{e} & U_{t}^{-1} \end{array}\right]\;\left[\begin{array}{c} b_{t}\\ \alpha_{t} \end{array}\right]=\left[\begin{array}{c} 0\\ {\bar{y}}_{t} \end{array}\right]$$

Then, we have:

(18) $$\begin{array}{c} b_{t}=\frac{e^{\text{T}}U_{t}y_{t}}{e^{\text{T}}U_{t}e}\\ \alpha_{t}=U_{t}\left(y_{t}-\frac{ee^{\text{T}}U_{t}y_{t}}{e^{\text{T}}U_{t}e}\right) \end{array}$$

As the number of snapshots increases, the dimension of Gramm matrix **Q***_i_* will be increasing because it is in proportional to the number of snapshots. Therefore, the computation for the regression parameters **α***_t_* and *b_t_* would be very intensive as the snapshots increase, and it is key issue for LS-SVR beamformer to find out a fast algorithm to improve the computation efficiency of **U***_i_*.

At time step *t*, **Q***_i_* and **H***_i_* are the matrixes with dimension of 2*N* × 2*N*:

(19) $$\begin{array}{c} Q_{t}=\left(\begin{array}{ccc} k({\bar{x}}_{1},{\bar{x}}_{1}) & \cdots & k({\bar{x}}_{t},{\bar{x}}_{1})\\ \vdots & \vdots & \vdots \\ k({\bar{x}}_{1},{\bar{x}}_{t}) & \cdots & k({\bar{x}}_{t},{\bar{x}}_{t}) \end{array}\right)\\ H_{t}=(Q_{t}+C^{-1}I)=\left(\begin{array}{ccc} k({\bar{x}}_{1},{\bar{x}}_{1})+1/C & \ldots & k({\bar{x}}_{2N},{\bar{x}}_{1})\\ \vdots & \ddots & \vdots \\ k({\bar{x}}_{1},{\bar{x}}_{2N}) & \cdots & k({\bar{x}}_{2N},{\bar{x}}_{2N})+1/C \end{array}\right) \end{array}$$

As time run to *t* + 1, new input snapshots **x***_t_*_+1_ and the corresponding desired array output *y_t_*_+1_ are added to the current training set. So **Q***_t_*_+1_ and **H***_t_*_+1_ can be represented as:

(20) $$\begin{array}{c} Q_{t+1}=\left(\begin{array}{cccc} k({\bar{x}}_{1},{\bar{x}}_{1}) & \ldots & k({\bar{x}}_{2N},{\bar{x}}_{1}) & k({\bar{x}}_{2(N+1)},{\bar{x}}_{1})\\ \vdots & \ddots & \vdots & \vdots \\ k({\bar{x}}_{1},{\bar{x}}_{2N}) & \cdots & k({\bar{x}}_{2N},{\bar{x}}_{2N}) & k({\bar{x}}_{2(N+1)},{\bar{x}}_{1})\\ k({\bar{x}}_{1},{\bar{x}}_{2(N+1)}) & \cdots & k({\bar{x}}_{2N},{\bar{x}}_{2(N+1)}) & k({\bar{x}}_{2(N+1)},{\bar{x}}_{2(N+1)}) \end{array}\right)\\ H_{t+1}=Q_{t+1}+C^{-1}I=\left(\begin{array}{cccc} k({\bar{x}}_{1},{\bar{x}}_{1})+1/C & \cdots & k({\bar{x}}_{2N},{\bar{x}}_{1}) & k({\bar{x}}_{2(N+1)},{\bar{x}}_{1})\\ \vdots & \ddots & \vdots & \vdots \\ k({\bar{x}}_{1},{\bar{x}}_{2N}) & \cdots & k({\bar{x}}_{2N},{\bar{x}}_{2N})+1/C & k({\bar{x}}_{2(N+1)},{\bar{x}}_{1})\\ k({\bar{x}}_{1},{\bar{x}}_{2(N+1)}) & \cdots & k({\bar{x}}_{2N},{\bar{x}}_{2(N+1)}) & k({\bar{x}}_{2(N+1)},{\bar{x}}_{2(N+1)})+1/C \end{array}\right) \end{array}$$

Comparing the elements of **H***_t_* and **H***_t_*_+1_, the matrix **H***_t_*_+1_ could be reconstructed by the matrix **H***_t_* plus an additional row and column, *i.e.*,

(21) $$H_{t+1}=\left(\begin{array}{cc} H_{t} & v_{t+1}\\ v_{t+1}^{\text{T}} & v_{t+1} \end{array}\right)$$

where, **v***_t_*_+1_ = [*k*(**x̄**_1_, (**x̄**_2(_*_N_*_+1)_), … *k*(**x̄**_2_*_N_*, (**x̄**_2(_*_N_*_+1)_)]^T^, *v_t_*_+1_ = *k*(**x̄**_2(_*_N_*_+1)_), **x̄**_2(_*_N_*_+1)_) + 1/*C*).

According to the theorem of inverting block matrix, the inverse of **H***_t_*_+1_ can be expressed by the inverse of **H***_t_* and the new column **v***_t_*_+1_ as:

(22) $$H_{t+1}^{-1}=\left(\begin{array}{cc} H_{t}^{-1}+\beta H_{t}^{-1}v_{t+1}v_{t+1}^{\text{T}}H_{t}^{-1} & -\beta H_{t}^{-1}v_{t+1}\\ -\beta v_{t+1}^{\text{T}}H_{t}^{-1} & \beta \end{array}\right)$$

where, $\beta =\left(v_{t+1}-v_{t+1}^{\text{T}}H_{t}^{-1}v_{t+1}\right)$. Thus the inverse of **H***_t_*_+1_,which is equal to **U***_t_*_+1_, can be calculated from the inverse of **H***_t_*, and it is not necessary to calculate the inverse of **H***_t_* when it has high dimension, so the computation complexity would be greatly reduced and the numerical stability problem arising from inverse matrix would be also avoided. When the set of snapshots is small, the **U***_t_* can be computed directly by matrix inverse theory.

## 5. Sparsification

The crucial drawback of LS-SVR beamformer is that it deals with high-dimension matrix, which is equal to the number of the snapshots due to the use of a quadratic constraint function. This would bring a big implementation problem to the proposed beamforming method since it is required to increase memory and computational resources as time evolves. Several methods have been proposed to cope with these problems [23,24]. The sliding-window approach [25] fixes the size of LS-SVR beamformer and allows it to be operated online in time-varying environments by keeping only the last *N* input snapshots in the sliding-window and simply abandoning those out of it. In [26], an exponential forgetting mechanism is introduced to describe the influence, which is imposed on the present situation by the past data [26]. This paper employs the novelty criterion, presented by Platt [27,28], to reduce the final size of the proposed beamformer, keep the algorithm complexity bounded and realize online sparsification. The basic idea of this approach is to construct a dictionary with center set *C* and update it appropriately according to the novelty criterion. The stages of the proposed specification are given as follows:

Step 1: Initialing an empty center set C_0_;
Step 2: Calculating the distance between the new snapshot x*_t_* and the present dictionary dis=min**_c_***_k_*_∈ C_*_i_* ‖**x***_t_* − **c***_k_*‖;
Step 3: If the distance obtained from Step 2 is smaller than the preset threshold *δ*_1_, x*_t_* is not added into the dictionary, otherwise the prediction error e*_i_* = *y_i_* − *ŷ_i_* is calculated;
Step 4: if |e|*_i_* is larger than another preset threshold *δ*_2_, x*_t_* is accepted as a new center and C*_i_* is updated to C*_i_*_+1_, otherwise go to Step 2.

Increasing *δ*_1_ and *δ*_2_, the final size of the LS-SVR beamformer will be decreased. But this will result to performance degradation. In practical applications, *δ*_1_ is set to around one tenth of the kernel bandwidth, and *δ*_2_ is around the square root of the steady-state mean square error (MSE). Cross-validation also can be used to select these appropriate thresholds.

Applying the above sparsification procedure, the computation complexity of the proposed beamformer will be reduced from *O*(*N*^2^) to *O*(*K*^2^), where *K* is the effective number of centers in the network at time *t*. As *K* is finite, the online real-time beamforming will be practical.

## 6. Simulation Tests

To evaluate the performance of the proposed LS-SVR-based beamformer, simulation tests are carried out. A 10 elements uniform linear array with half-wavelength spacing is taken into account. The desired signal comes from a presumed direction *θ* = 3° and two irrelevant interferences, with interference-to-noise ratio (INR) of 20 dB, impinge on the array from *θ*_2_ = −32° and *θ*_3_ = 17° respectively. The additive noise is assumed to be a 0-dB complex white Gaussian distributed random variable. For comparison purpose, the conventional MVDR, the diagonal loading MVDR (MVDR-DL), the ES [29], the SQP [9] and the RR [30] method are considered. The parameters of the proposed beamformer, σ, *δ*_1_ and *δ*_2_, are chosen as 1.0, 0.1 and 0.08 respectively. The load value of MVDR-DL beamformer is set to (*P_e_*+10 *dB*), where *P_e_* denotes the power of desired signal. All results are obtained from 100 independent simulation runs.

**Figure 1.**
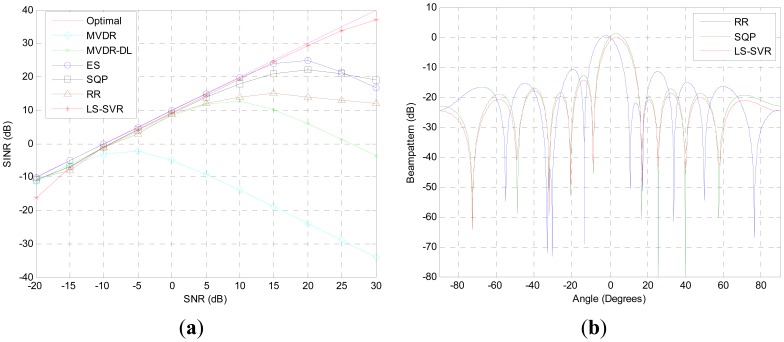
Scenario with only DOA mismatch (**a**) Output SINR versus SNR; (**b**) Beam-patterns, SNR = 10 dB, 2 interference.

The first simulation aims to compare the performance of these beamformers when steering vector mismatch is presented. From Figure 1(a), we observe that the proposed LS-SVR beamformer consistently improves its output SINR as SNR increases and performs much closer as the idea one when the input SNR is varied from −20 dB to 30 dB. Due to the DOA mismatch, the interested signal is considered as interference and a null is allocated in the desired signal direction by the MVDR beamformer. As a result, the output SINR is decreased. When input SNR is larger than −5 dB, the output SINR of MVDR beamformer degrades seriously. In comparing with the MVDR beamformer, the MVDR-DL, ES, SQP and RR methods get more robustness against DOA mismatch. But they still suffer from a degradation of performance while the input SNR becomes higher.

Figure 1(b) shows the normali\zed beampattern plots when the input SNR is equal to 10 dB. As it is illustrated, all beam-patterns of the robust beamformers have nulls at the DOAs of the interferences. But the proposed LS-SVR still outperforms others by markedly lower sidelobe level, and maintaining distortionless response for the desired signal.

**Figure 2.**
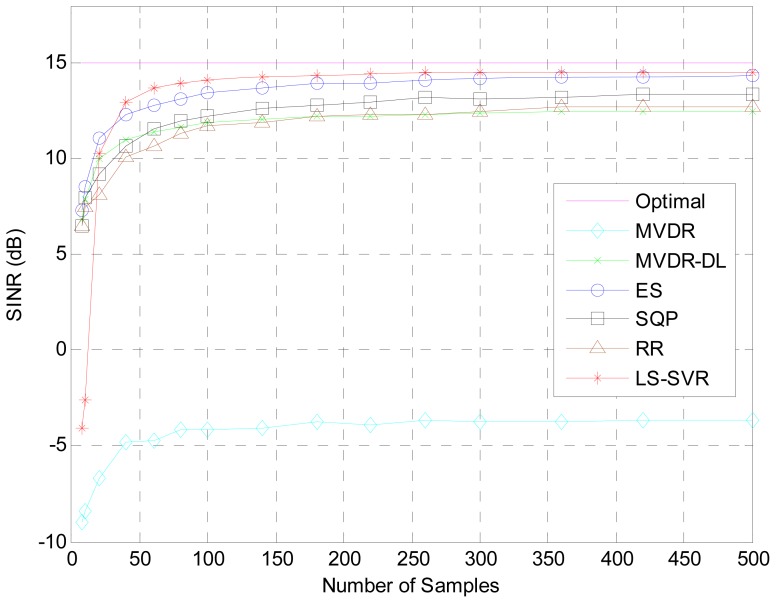
Scenario with limited snapshots and two interferences.

The covariance matrix would be inaccurately estimated owing to insufficient snapshots, DOA mismatch of desired signal and array calibration errors. This kind of inaccuracy may result in the degradation of array response. Hence, both the errors of insufficient snapshots and DOA mismatch are considered to verify the proposed beamformer in our second simulation tests. Figure 2 shows the resulting output SINRs versus the snapshot number *K*. When snapshots are over 20, the LS-SVR clearly outperforms other beamformers tested. Owing to the steering vector mismatch, the MVDR beamformer see the desired signal as interference and fails in its operation.

**Figure 3.**
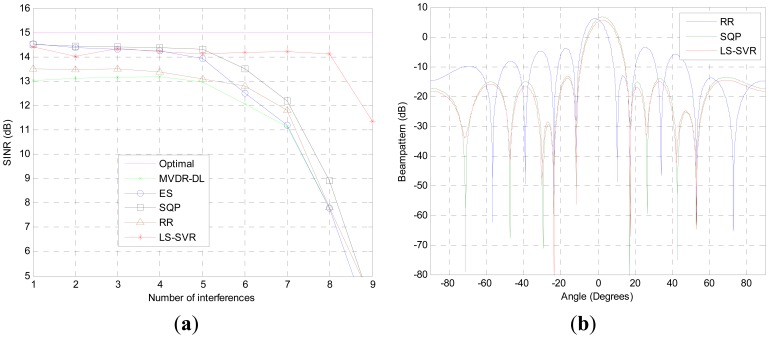
Scenario with DOA mismatch and multiple interferences (**a**) SINR versus Number of interferences; (**b**) Beam-patterns, SNR = 10 dB, 4 interference.

The performance of the proposed beamformer in the scenario with multiple interferences is demonstrated in the third test. The steering vector mismatch is also presented. As it can be seen from Figure 3(a), the proposed algorithm performs equally well as ES and SQP when the number of interferences less than 5. When the interference numbers is increased to 8, the output SINR of the proposed LS-SVR beamformer is only 1 dB lower than that of idea beamformer. In contrast, the output SINRs of other beamformers tested are dramatically decreased due to the decrease of the available freedom degrees which are devoted to suppress the interference.

The corresponding beampatterns of the beamformers are demonstrated in Figure 3(b), where the four interferences with DOAs of *θ_i_* = [17.4°, −11.5°, 53.1°, −23.5°] are taken into account. It can be seen that the LS-SVR beamformer not only presents deep nulls at the DOAs of interference, but also achieves better sidelobe suppression than other beamformers tested. Thus, the proposed LS-SVR method can get better SINR performance than the usual robust linear beamforming algorithms in the case of numerous interferences.

**Figure 4.**
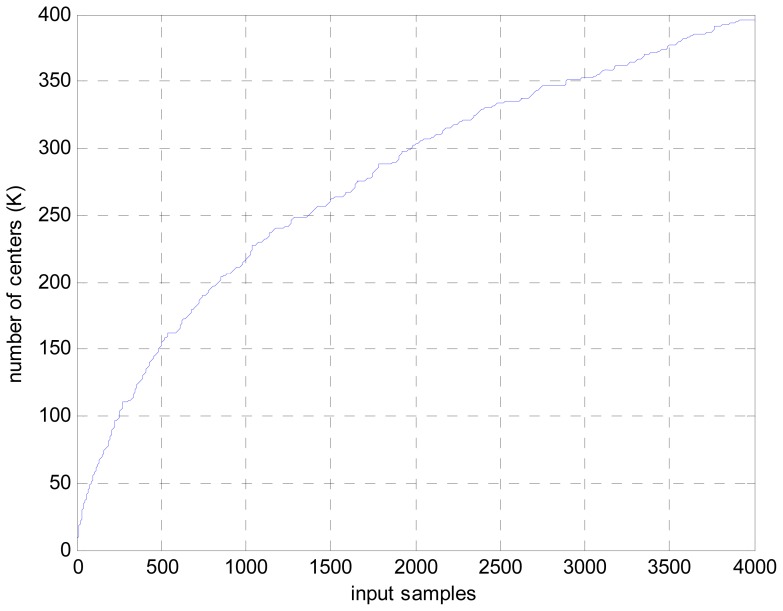
Dictionary size *vs.* input samples for the novel approach.

To show the computation complexity of the novel approach, the dictionary size growth with the input samples is given in Figure 4. As it can be seen in Figure 4, only 396 center numbers are needed to calculate the beamformed output for 4,000 input samples. In comparison with the original LS-SVR algorithm, in which 4,000 centers are needed for the same case. Thus, the computation cost is largely reduced.

## 7. Conclusions

We present a novel nonlinear LS-SVR-based beamforming approach in this paper. This approach first uses a squared-loss function to replace the conventional linearly constrained minimum variance cost function, which can significantly increase robustness against mismatch problems and provide additional control over the sidelobe level. The method also applies Gaussian kernels to the array observations to improve the generalization capacity. Finally, the method uses a recursive regression procedure to estimate the weight vectors on real-time and performs mode reduction to reduce the final size of the beamformer.

The simulation tests, with steering vector mismatch, numerous interferences and limited available snapshots, are carried out to verify the performance of the proposed beamforming algorithm in comparison with other recently proposed ones. The test results show that the proposed beamforming method significantly outperforms many other recently proposed linear robust beamforming techniques in terms of signal distortion in the desired signal and noise reduction in scenarios with DOA mismatch, limited observation samples, and numerous interferences.

## References

[1] Van Trees H.L. (2002). Part IV Detection, Estimation and Modulation Theory. Optimum Array Processing.

[2] Liu J., Gershman A.B., Luo Z.Q., Wong K.M. (2003). Adaptive beamforming with sidelobe control: A second-order cone programming approach. IEEE Signal Process. Lett..

[3] Li J., Stoica P., Wang Z. (2004). Doubly constrained robust Capon beamformer. IEEE Trans. Signal Process.

[4] Li J., Stoica P. (2006). Robust Adaptive Beamforming.

[5] Liu C., Liao G. (2010). Robust capon beamformer under norm constraint. Signal Process.

[6] Li J., Stoica P., Wang Z. (2003). On robust capon beamforming and diagonal loading. IEEE Trans. Signal Process.

[7] Lorenz R.G., Boyd S.R. (2005). Robust minimum variance beamforming. IEEE Trans. Signal Process.

[8] Vorobyov S.A., Gershman A.B., Luo Z.Q. (2003). Robust adaptive beamforming using worst-case performance optimization: A solution to the signal mismatch problem. IEEE Trans. Signal Process.

[9] Hassanien A., Vorobyov S.A., Wong K.M. (2008). Robust adaptive beamforming using sequential quadratic programming: An iterative solution to the mismatch problem. IEEE Signal Process. Lett..

[10] Lie J.P., Ser W., See C.M. (2011). Adaptive uncertainty based iterative robust capon beamformer using steering vector mismatch estimation. IEEE Trans. Signal Process.

[11] Landau L., de, Lamare R.C., Haardt M. Robust Adaptive Beamforming Algorithms Using Low-Complexity Mismatch Estimation.

[12] Yu Z.L., Er M.H. (2006). A robust minimum variance beamformer with new constraint on uncertainty of steering vector. Signal Process.

[13] Nai S.E., Ser W., Yu Z.L., Rahardja S. (2009). A robust adaptive beamforming framework with beampattern shaping constraints. IEEE Trans. Antennas Propagat.

[14] Yu Z.L., Ser W., Er M.H., Gu Z., Li Y. (2009). Robust adaptive beamformers based on worst-case optimization and constraints on magnitude response. IEEE Trans. Signal Process.

[15] Martinez-Ramon M., Rojo-Alvarez J.L., Camps-Valls G., Christodoulou C.G. (2007). Kernel antenna array processing. IEEE Trans. Antennas Propagat.

[16] Chang P.R., Yang W.H., Chan K.K. (1992). A neural network approach to MVDR beamforming problem. IEEE Trans. Antennas Propagat.

[17] Vapnik V.N. (1995). The Nature of Statistical Learning Theory.

[18] Gaudes C.C., Santamaria I., Via J., Gomez E.M.M., Paules T.S. (2007). Roubust array beamforming with sidelobe control using support vector machines. IEEE Trans. Signal Process.

[19] Ramon M.M., Xu N., Christodoulou C.G. (2005). Beamforming using support vector machines. IEEE Antenn. Wirel. Propag. Lett..

[20] Suykens J.A.K., De Brabanter J., Lukas L., Vandewalle J. (2002). Weighted least squares support vector machines: Robustness and sparse approximation. Neurocomputing.

[21] Chu W., Ong C.J., Keerthi S.S. (2005). An improved conjugate gradient scheme to the solution of least squares svm. IEEE Trans. Neural Networks.

[22] Adankon M.M., Cheriet M. (2009). Model selection for LSSVM application to handwriting recognition. Pattern Recognit.

[23] Hoegaerts L., Suykens J.A.K., Vandewalle J., de Moor B. (2005). Subset based least squares subspace regression in RKHS. Neurocomputting.

[24] Jiao L., Bo L., Wang L. (2007). Fast sparse approximation for least square support vector machine. IEEE Trans. Neural Networks.

[25] Vaerenbergh S.V., Via J., Santamaria I. A Sliding-Window Kernel RLS Algorithm and Its Application to Nonlinear Channel Indentification.

[26] Slavakis K., Theodoridis S., Yamada I. (2008). On line classification using kernels and projection based adaptive algorithm. IEEE Trans. Signal Process.

[27] Platt J. (1991). A resource allocating network for function interpolation. Neural Comput..

[28] Liu W.F., Jose C.P., Simon H. (2010). Kernel Adaptive Filtering.

[29] Yu J.L., Yeh C.C. (1995). Generalized eigenspace-based beamformers. IEEE Trans. Signal Process.

[30] Selen Y., Abrahamsson R., Stoica P. (2008). Automatic robust adaptive beamforming via ridge regression. Signal Process.

